# Normal interventricular differences in tissue architecture underlie right ventricular susceptibility to conduction abnormalities in a mouse model of Brugada syndrome

**DOI:** 10.1093/cvr/cvx244

**Published:** 2017-12-18

**Authors:** Allen Kelly, Simona Salerno, Adam Connolly, Martin Bishop, Flavien Charpentier, Tomas Stølen, Godfrey L Smith

**Affiliations:** 1 Department of Circulation and Medical Imaging, St Olav’s Hospital, Norwegian University of Science and Technology (NTNU), Postboks 8905, 7491 Trondheim, Norway; 2 Institute of Cardiovascular & Medical Sciences, University of Glasgow G12 8QQ, UK; 3 Division of Imaging Sciences and Biomedical Engineering, Department of Biomedical Engineering, Kings College London SE1 7EH, UK; 4 L'Institut du Thorax, Inserm, CNRS, UNIV Nantes 44000, France

**Keywords:** Brugada syndrome, Right ventricular structure, Sodium channel, Multiphoton microscopy

## Abstract

**Aims:**

Loss-of-function of the cardiac sodium channel Na_V_1.5 is a common feature of Brugada syndrome. Arrhythmias arise preferentially from the right ventricle (RV) despite equivalent Na_V_1.5 downregulation in the left ventricle (LV). The reasons for increased RV sensitivity to Na_V_1.5 loss-of-function mutations remain unclear. Because ventricular electrical activation occurs predominantly in the transmural axis, we compare RV and LV transmural electrophysiology to determine the underlying cause of the asymmetrical conduction abnormalities in Scn5a haploinsufficient mice (*Scn5a^+/−^*).

**Methods and results:**

Optical mapping and two-photon microscopy in isolated-perfused mouse hearts demonstrated equivalent depression of transmural conduction velocity (CV) in the LV and RV of *Scn5a^+/−^* vs. wild-type littermates. Only RV transmural conduction was further impaired when challenged with increased pacing frequencies. Epicardial dispersion of activation and beat-to-beat variation in activation time were increased only in the RV of *Scn5a*^+/−^ hearts. Analysis of confocal and histological images revealed larger intramural clefts between cardiomyocyte layers in the RV vs. LV, independent of genotype. Acute sodium current inhibition in wild type hearts using tetrodotoxin reproduced beat-to-beat activation variability and frequency-dependent CV slowing in the RV only, with the LV unaffected. The influence of clefts on conduction was examined using a two-dimensional monodomain computational model. When peak sodium channel conductance was reduced to 50% of normal the presence of clefts between cardiomyocyte layers reproduced the activation variability and conduction phenotype observed experimentally.

**Conclusions:**

Normal structural heterogeneities present in the RV are responsible for increased vulnerability to conduction slowing in the presence of reduced sodium channel function. Heterogeneous conduction slowing seen in the RV will predispose to functional block and the initiation of re-entrant ventricular arrhythmias.

## 1. Introduction

The cardiac sodium channel (Na_V_1.5) is responsible for the rapid upstroke phase of the action potential (AP) in both atrial and ventricular myocytes. Consequently, this protein plays a critical role in regulating cell excitability and electrical propagation in the heart. Mutations in *SCN5A*, the gene that encodes the α-subunit of Na_V_1.5, have been linked to a variety of inherited cardiac arrhythmic disorders.^1^ The Scn5a haploinsufficient mouse model (Scn5a^+/^^−^) has emerged as a powerful tool to study arrhythmia syndromes associated with loss-of-function Na-channel mutations,^2^ in particular Brugada syndrome (BrS), which is characterized by a risk of sudden cardiac death (SCD) due to ventricular fibrillation (VF) in patients with structurally normal hearts^3^ and poses a significant challenge for clinicians.

In BrS, arrhythmias arise preferentially from the right ventricle (RV), particularly the RV outflow tract (RVOT),^4^^,^^5^ although the RV free wall itself may be more prone to arrhythmias. Scn5a^+/^^−^ mice exhibit increased effective refractory periods (RV-specific) coupled with abnormal restitution and AP alternans.^6^ In addition, transmural gradients of repolarization in the RV have been reported in this model, which are increased by the Na-channel blocker flecainide (which is pro-arrhythmic in BrS patients) and reduced by the antiarrhythmic agent quinidine (which is effective in BrS).^7^^,^^8^ Debate remains ongoing as to the underlying ionic mechanisms responsible for this electrical phenotype.^9^ Transmural gradients in Na_v_1.5 and K_v_4.2/K_v_4.3 (responsible for fast sodium [I_Na_] and transient outward potassium [I_to_] currents, respectively) are known to exist in both LV and RV of several species. This produces distinct cellular electrical phenotypes between myocytes isolated from the subendocardium and subepicardium, creating the potential for transmural gradients of repolarization.^10^ These differences in isolated cells are believed to contribute to the RV origin of arrhythmias in *Scn5a*^+/^^−^ mice^11^ and in a pharmacological model of BrS in the canine ventricular wedge preparation.^12^^,^^13^

Problems arise with this view, however, due to the strong electrotonic interaction between well-coupled myocytes in the intact myocardium, which mitigates much of the spatial difference in intrinsic AP duration, even when accounting for gradients in I_to_ expression.^14^ This is particularly important in smaller species such as the mouse, as functional AP duration gradients are dependent upon physical tissue size. As such, while myocytes isolated from the LV and RV in models of Na-channel dysfunction appear electrophysiologically distinct, these cellular differences alone may not be enough to explain the increased propensity to RV arrhythmias in the intact heart. Understanding of the tissue-level requirements for ventricular arrhythmia is crucial for the development of novel risk stratification and treatment pathways for patients with loss-of-function Na-channel mutations. We therefore set out to identify the key interventricular differences in transmural electrophysiology and structure, which could explain increased arrhythmogenesis in the RV of Scn5a^+/^^−^ mice. Using optical mapping combined with two-photon (2 P) microscopy to analyse surface and transmural electrical propagation in the isolated perfused mouse heart, we demonstrate that substantial differences in transmural conduction exist between the LV and RV of Scn5a^+/^^−^ mice. These differences did not arise from increased interstitial fibrosis but rather from normal structural discontinuities present in the RV, but not the LV wall. Selective pharmacological downregulation of I_Na_ using tetrodotoxin (TTX) in hearts from young wild-type (WT) mice recapitulated the RV-specific transmural conduction defects seen in Scn5a^+/^^−^ mice. Computational modelling indicated the key role of non-conducting clefts in evoking activation heterogeneity and transmural dispersion of activation through tortuous conduction pathways when Na channel conductance (gNa) was reduced. Thus, the normal physiological differences in transmural tissue architecture between the RV and LV are major contributors to the RV bias of electrical dysfunction in conditions characterized by loss-of-function of the Na-channel.

## 2. Methods

### 2.1 Ethical statement

All *in vivo* experiments were performed in the animal facility of Nantes University Health Research Institute (Unité de Thérapeutique Expérimentale), which has been accredited by the French Ministry of Agriculture. Experimental procedures were approved by the regional ethics committee (Comité d’éthique en expérimentation animale—Pays de la Loire). *Ex vivo* whole heart experiments were approved by the Norwegian Council for Animal Research and were conducted in accordance with the Guide for the Care and Use of Laboratory Animals (National Institutes of Health, 8th edition, revised 2011).

### 2.2 Animal genotyping and electrocardiography

*Scn5a* ^+/^^−^ mice with a 129/Sv genetic background were genotyped by polymerase chain reaction as previously described.^15^ Mice were anaesthetized for electrocardiogram (ECG) recording with etomidate (25 mg/kg, i.p.). Body temperature was maintained at 37 °C with a heating pad (Harvard Apparatus, Holliston, MA, USA). Six-lead ECG was recorded with 25-gauge subcutaneous electrodes on a computer through an analogue–digital converter (IOX 1.585; EMKA Technologies, Paris, France) for monitoring and subsequent analysis (ECG Auto v3.2.0.2; EMKA Technologies). The ECGs were analyzed as previously described.^15^

### 2.3 Isolated heart preparation

Initially, 11 *Scn5a*^+/^^−^ mice (63.9 ± 1.8 weeks) and 10 WT littermates (62.2 ± 2.4 weeks) were used in isolated perfused heart experiments. Animals were sedated with a gaseous mixture of 2% isoflurane in 70:30 N_2_O and O_2_ and administered an intravenous injection of heparin (1 ml of a 5000-IU solution per kg body weight) to minimize clotting. The excised hearts were cannulated and underwent retrograde perfusion with oxygenated 37 °C Tyrode’s solution containing (in millimole per liter) NaCl (116.0), NaHCO_3_ (20.0), Na_2_HPO_4_ (1.0), MgSO_4_ (1.0), KCl (5.0), CaCl_2_ (1.5), Glucose (11.0), Na pyruvate (1.2)—pH 7.4 maintained by continuous bubbling with 95% O_2_/5% CO_2_ gas mixture. Flow was adjusted to yield a coronary perfusion pressure of 60–80 mmHg. Heart rate was monitored throughout by a pseudo-ECG recording. Contraction was suppressed with continuous perfusion of blebbistatin (10 µmol/l) and 2, 3-butanedione monoxime (5 mmol/l). Hearts were loaded with the fluorescent indicator di-4-ANEPPS (25 µl of 2 mmol/l stock solution) for voltage measurements. A schematic of the isolated heart setup is shown in Supplementary material online, *Figure S1A*.

### 2.4 Optical AP recordings using widefield epifluorescence and 2P microscopy

Equipment and setup is shown in Supplementary material online, *Figure S1B* and has been described previously.^16^ Briefly, a 128×128 pixel CardioCMOS-SM128 camera and 120 fs pulsed Ti-sapphire laser were mounted to a 2 P microscope system (Intelligent Imaging Innovations, Denver, CO, USA) for widefield (WF) optical mapping and transmural multiphoton imaging, respectively. The WF voltage mapping was performed via a 2.5X, 0.3 numerical aperture (NA) objective lens (470 nm LED light source) at 5 kHz. Imaging was switched to a 20X 1.0 NA lens for high resolution transmural imaging with 2 P. Transmural line scans (2.5 kHz) between 450  and 50 µm beneath the epicardial surface (in 50 µm steps) captured AP data (920 nm excitation). Initially, four hearts were stimulated at a 7 Hz basic frequency from either the right atrium (RA) or the LV endocardial surface at the apex. Once it was established that ventricular transmural conduction velocity (CV) was not different between these two pacing sites (summarized in Supplementary material online*, Figure S2*), pacing was performed exclusively at the LV endocardial apex. Hearts from three *Scn5a^+/^*^*−*^ mice were unable to tolerate the baseline pacing frequency of 7 Hz used throughout the study, with no signs of ischemia or perfusion issues. The maximum pacing frequency tolerated by these hearts was recorded (see *Figure 2C*) after which they were excluded from all subsequent analysis. Data were collected while hearts were paced at 7 Hz initially and then the highest stable pacing frequency possible, defined as the highest frequency where 1:1 capture could be maintained. Trains of 20 APs from each line scan recording were averaged relative to the pacing stimulus pulse to achieve a single transient. No further data filtering was necessary. This allowed temporal information of the transient, particularly from the upstroke, to be preserved. This approach has been verified previously in hearts from several small animal species, including mouse.^17^

### 2.5 Histological staining

Following the optical experiments, a subset of hearts (WT; *n *= 6; *Scn5a^+/^*^*−*^, *n* = 5) were fixed in a freshly prepared 4.0% solution of paraformaldehyde and stored at 4 °C overnight, before being transferred to a 0.4% solution of paraformaldehyde until paraffin embedding. Paraffin-embedded tissue was then sectioned into 4 µm-thick slices (eight non-consecutive slices per heart; 20 µm gap between each slice) at the level of the ventricular midwall using a Leica RM2235 microtome (Leica Biosystems, Wetzlar, Germany). Cardiac fibrosis was detected by staining slices with Masson’s trichrome. A single value both for fibrosis and non-vascular cleft quantification for the free midwall of each heart was reported from the mean of the eight non-consecutive short-axis slices to obtain a representative estimate of cleft size from the intramural region examined using 2 P.

### 2.6 Computational modelling

Tissue dynamics were simulated using the Cardiac Arrhythmia Research Package^18^ in a two-dimensional rectangular region of 0.8 × 5 mm (approximate transmural width of the RV free wall). The mono-domain equation was solved using a spatially uniform quadrilateral mesh (10 µm edge length), with rectangular clefts (represented as internal boundaries) of size (t × w µm) and orientation (*θ* radians), chosen from a uniform distribution and arranged randomly in the domain. The Bondarenko mouse ionic model was used to represent ionic membrane dynamics with a global stable time-step of 5µs.^19^ The single-cell model was paced for 100 beats to reach a steady state before use within the tissue model. Fibres were aligned with the *x*-axis and the diagonal terms of the conductivity tensor were (sigma_xx, sigma_yy) = (0.13, 0.02)S/m. Stimuli were applied to nodes along the (endo) *y* = 0 line and average measurements were made along the (epi) *y* = 800 µm line. To numerically represent electrophysiology of the *Scn5a*^+/^^−^ myocytes, the maximum sodium channel conductivity (*normal* gNa) was reduced by a factor of two (*low* gNa). The APs were also generated from single-cell simulations using the Bondarenko model with and without lowered gNa.

### 2.7 Experimental blinding

Isolated heart experimenters were blinded to the genotype of the mouse by the technician responsible for animal sacrifice. A separate blinding procedure was used once hearts were preserved for histology. Operators performing either tissue histology image analysis or 2 P image analysis, were unblinded only after each data set was fully analysed. Due to timing of isolated heart experiments relative to histological tissue preparation and analysis, no blinding of RV/LV conduction and structure correlation was possible.

### 2.8 Data analysis

The 10% point of the AP rising phase relative to stimulus pulse was used as an index of tissue activation at each transmural layer, allowing calculation of transmural CV and beat-to-beat activation variability. AP duration (APD) was calculated at 30, 50, and 80% repolarization levels. WF and 2 P data were analysed using custom written software in Delphi (Borland, Austin, TX, USA) and Matlab (Mathworks, Natick, MA, USA), respectively. Data are expressed as mean ± standard error. Statistical analysis was performed in Graphpad prism 7 (La Jolla, CA, USA) using Student’s *t-*test (paired where appropriate), two-way analysis of variance (ANOVA) or repeated measures two-way ANOVA with Bonferroni multiple comparisons test, as indicated in figure legends. *P* <0.05 were considered significant.

## 3. Results

### 3.1 Baseline AP characteristics of Scn5a^+/^^−^ hearts

**Figure 1:**
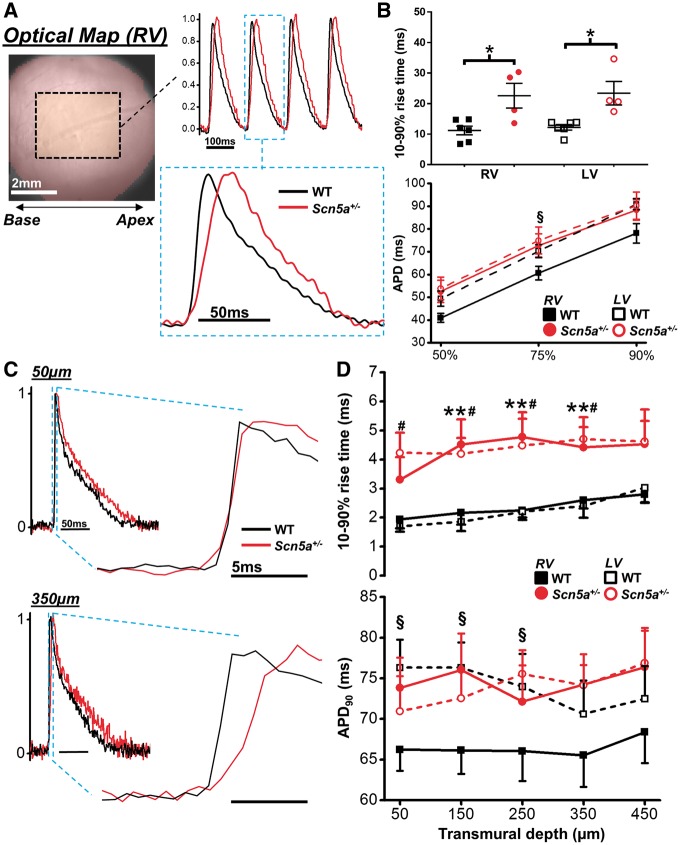
**  Epicardial and transmural electrophysiology of *Scn5a^+/-^* mouse hearts.** (*A*) Example optical mapping action potentials (APs) from the right ventricle of wild type (WT - black) and *Scn5a^+/-^* (red) hearts. (*B*) **upper panel,** mean AP rise time was significantly longer in hearts from *Scn5a^+/-^* mice compared to WT mice. Lower panel, AP duration at 50, 75, and 90% repolarization levels from the left ventricle (LV) and RV in WT and *Scn5a^+/-^* hearts. (*C*) Example AP traces from RV 2 P line scans at 50 µm (upper) and 350 µm (lower) below the epicardial surface (black line = WT; red line = *Scn5a*^+/−^). Expanded section highlights AP upstroke**.** (*D*) Mean data of AP rise time (upper) and AP duration at progressively deeper transmural layers from the RV (closed symbols, solid lines) and LV (open symbols, dashed lines) in WT (black squares—*n* = 10) and *Scn5a^+/−^* (red circles—*n* = 9) hearts. *P <* *0.05, *Scn5a^+/−^* vs. WT; **P <* *0.05, RV *Scn5a^+/-^* vs. RV WT; ^#^*P *<* *0.05, LV *Scn5a^+/−^* vs. LV WT; ^§^*P *<* *0.05, RV vs. LV (WT). Two-way ANOVA with Bonferroni correction for multiple comparisons for panels in B, paired and unpaired student’s *t*-test used for comparison between ventricles and between WT and *Scn5a^+/−^*, respectively (panels in D).

The electrophysiological defects of the *Scn5a*^+/^^−^ model were initially quantified via ECG, recorded *in vivo* at 10 weeks of age in all animals. Representative examples are shown in Supplementary material online, *Figure S3 A*. *Scn5a^+/^*^*−*^ mice exhibited marked slowing of ventricular conduction, characterized by a prolonged QRS duration compared with WT mice (see Supplementary material online, *Figure S3B—*left). ECG records from isolated perfused hearts in sinus rhythm confirmed the continued presence of this electrophysiological deficit in the *Scn5a*^+/^^−^ vs. WT mice (see Supplementary material online, *Figure S3B—*right). Baseline electrophysiology was then explored in more detail using optical mapping in a subset of isolated perfused hearts (WT; *n* = 6, *Scn5a^+/^*^*−*^; *n* = 4). Slower AP rise times were found across the region of interest in the RV of *Scn5a*^+/^^*−*^ vs. WT mouse hearts (*Figure 1A* and *B*—top panel). AP duration in the RV of *Scn5a^+/^*^*−*^ hearts was also found to be significantly longer vs. WT RV at 75% repolarization (*Figure 1B—*lower panel). To identify specific intramural features of the conduction defect in the *Scn5a^+/^*^*−*^ mice, transmural electrophysiology was investigated using sequential line scan recordings through the LV and RV wall. Typical averaged APs from WT (black trace) and *Scn5a*^+/^^−^ (red trace) mouse hearts are shown in *Figure 1C* from the sub-epicardium (top) and mid-myocardium (bottom) of the RV. Line-scan recordings with 2 P revealed that AP rise time (*Figure 1D—*upper panel) was similarly slowed in the LV and RV of *Scn5a*^+/^^−^ hearts (dashed lines) throughout the transmural region measured, compared with WT hearts (solid lines). WT LV exhibited significantly longer APDs throughout the epicardial and sub-epicardial regions compared with RV, but this interventricular difference was abolished in *Scn5a^+/^*^*−*^ hearts (*Figure 1D—*lower panel). No transmural gradient of APD was detected in the LV or RV of either WT or *Scn5a^+/^*^*−*^ hearts.

### 3.2 Transmural conduction

**Figure 2:**
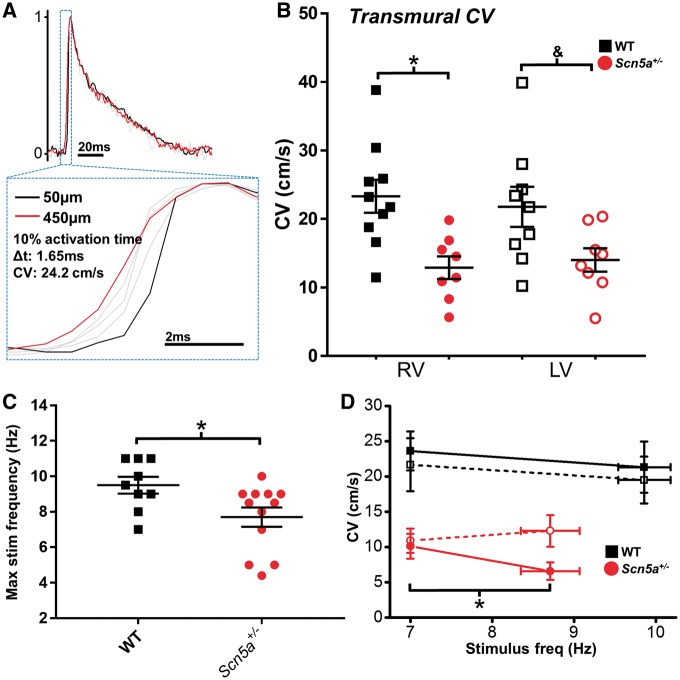
**  Transmural conduction differences between LV and RV.** (*A*) Transmural CV was measured as the difference in activation time (time at 10% AP upstroke) between 450  and 50 µm below the epicardial surface. (*B*) Transmural CV in the RV (closed symbols) and LV (open symbols) at 7 Hz pacing frequency from WT (black squares, *n* = 10) and *Scn5a^+/−^* (red circles, *n* = 8) hearts. (*C*) Scatter plots showing the range of the fastest stable pacing frequency in WT (black squares, *n* = 10) and *Scn5a^+/−^* (red circles, *n* = 12) hearts. Stable pacing was defined as 1:1 capture on electrocardiogram records. (*D*) Change in transmural CV in the RV (closed symbols) and LV (open symbols) at baseline pacing frequency (7 Hz) and fastest stable pacing frequency, in WT (black squares, *n* = 9) and *Scn5a^+/−^* (red circles, *n* = 6) hearts. B *P <* *0.05 and ^&^*P *=* *0.05, WT vs. *Scn5a^+/-^* (two-way ANOVA with Bonferroni multiple comparison’s post-test); C *P <* *0.05, WT *vs Scn5a^+/−^* (unpaired Student’s *t*-test); D *P <* *0.05, baseline vs. rapid pacing (paired Student’s *t*-test).

Defects in transmural conduction leading to conduction block play a major role in the appearance of arrhythmias in Na channel loss-of-function mutations. To better understand the relative impact of Na channel abundance on LV and RV transmural conduction, 2 P line-scans at each epicardial layer were time-matched relative to the endocardial stimulus pulse, and inter-layer CV calculated using the time delay between layer activation and distance of electrical propagation (illustrated in *Figure 2A*). At 7 Hz, CV in both ventricles was lower in *Scn5a*^+/^^−^ hearts compared to WT (LV; *P *=* *0.05, *n* = 8), and no significant difference was present between ventricles (*Figure 2B*). When transmural conduction was challenged, however, by gradually increasing the stimulation frequency, the maximal rate displaying 1:1 ventricular capture was significantly lower in the *Scn5a*^+/^^−^ hearts than in the WT hearts (*Figure 2C*). Crucially, at these maximal stimulus frequencies, CV was significantly reduced in the RV but not LV of *Scn5a*^+/^^−^ hearts, whereas CV in the WT hearts was not significantly altered using maximal stimulus frequencies in either ventricle (*Figure 2D*).

### 3.3 Ventricular activation characteristics

**Figure 3:**
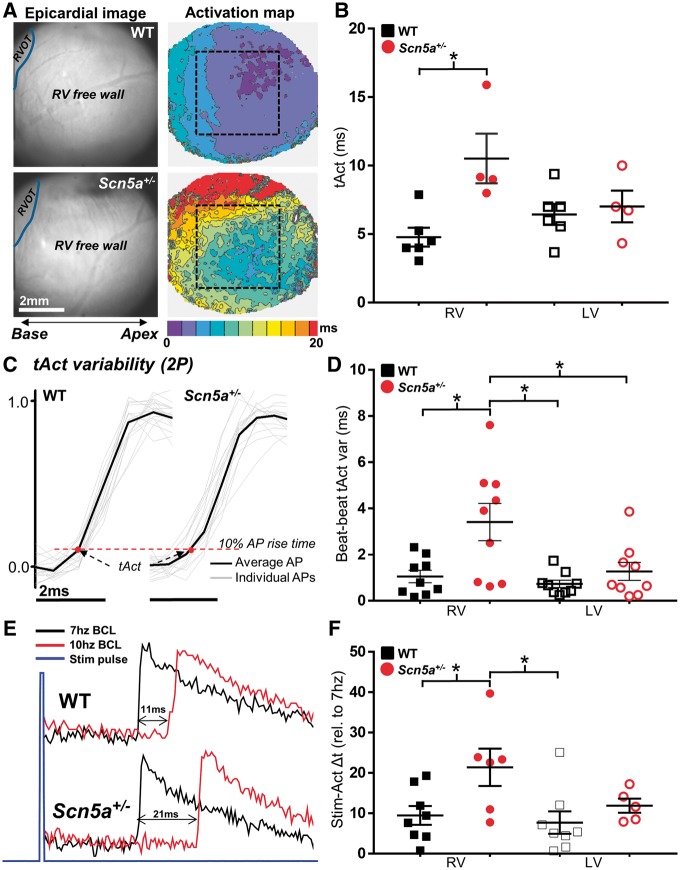
**  Ventricular spatial and temporal activation characteristics in *Scn5a^+/−^* mice.** (*A*) Widefield (WF) epicardial activation maps obtained from the RV surface of WT (upper panel) and *Scn5a^+/−^* (lower panel) mouse hearts. Region of interest indicated by dashed black box. (*B*) Mean epicardial activation times (tAct) from WF images for each ventricle, in WT (black squares, *n* = 6) and *Scn5a^+/−^* (red circles, *n* = 4) hearts. (*C*) Individual traces of AP upstrokes from WT (left) and *Scn5a^+/−^* hearts (right) recorded using 2 P excitation, time matched to the electrical stimulus pulse. Black line indicates the temporally averaged AP resultant from individual traces. Measured tAct (time from stimulus to 10% of AP rise time) is indicated by a red dot. (*D*) Mean beat-to-beat AP activation variability (tAct var) for both ventricles, in WT (black squares, *n* = 9) and *Scn5a^+/−^* (red circles, *n* = 9) hearts. (*E*) Example 2 P line scan traces showing increased activation delay in the RV when pacing frequency is increased. (*F*) Difference in AP stimulus-activation delay (Stim-Act Δt) at highest stable pacing frequency relative to 7 Hz pacing. Panels B, D, and F *P <* *0.05; comparisons made using two-way ANOVA with Bonferroni multiple comparisons post-test. RVOT; Right ventricular outflow tract.

This RV-specific conduction defect was studied in more detail by examining electrical activation characteristics using both WF and 2 P. *Figure 3A* illustrates the activation maps obtained from WF optical mapping of the ventricular epicardium. Typical RV camera images and the corresponding fluorescence activation time maps are shown for WT (upper) and *Scn5a*^+/^^−^ (lower) hearts, illustrating significant slowing and dispersion of activation in the RV of *Scn5a^+/^*^*−*^ hearts. The time for 95% of whole-field activation was significantly longer in the RV of *Scn5a*^+/^^−^ hearts vs. WT hearts; however, no between-group difference was found for LV (*Figure 3B*). Variability in activation time (tAct) on a beat-to-beat basis was assessed in the epicardial layers from a sequence of 25 APs acquired using 2 P excitation. Representative AP upstrokes recorded using this method are overlaid with respect to the timing of the electrical stimulus pulse in *Figure 3C* (grey lines). Once again, *Scn5a*^+/^^−^ hearts showed a significantly increased variability in tAct preferentially in the RV but not the LV in comparison to WT hearts (*Figure 3D*). Differential RV activation behaviour in *Scn5a*^+/^^−^ hearts was also observed upon increasing stimulation frequency. *Figure 3E* shows the relative change in time between stimulus and epicardial activation upon increasing stimulation frequency from 7 Hz to maximum stable frequency (10 Hz in the example trace). The increased pacing frequency resulted in a significantly increased stimulus to activation delay in the RV of *Scn5a*^+/^^−^ hearts relative to WT hearts, but no observed difference in LV (*Figure 3F*).

### 3.4 Structural characterization of LV and RV

**Figure 4:**
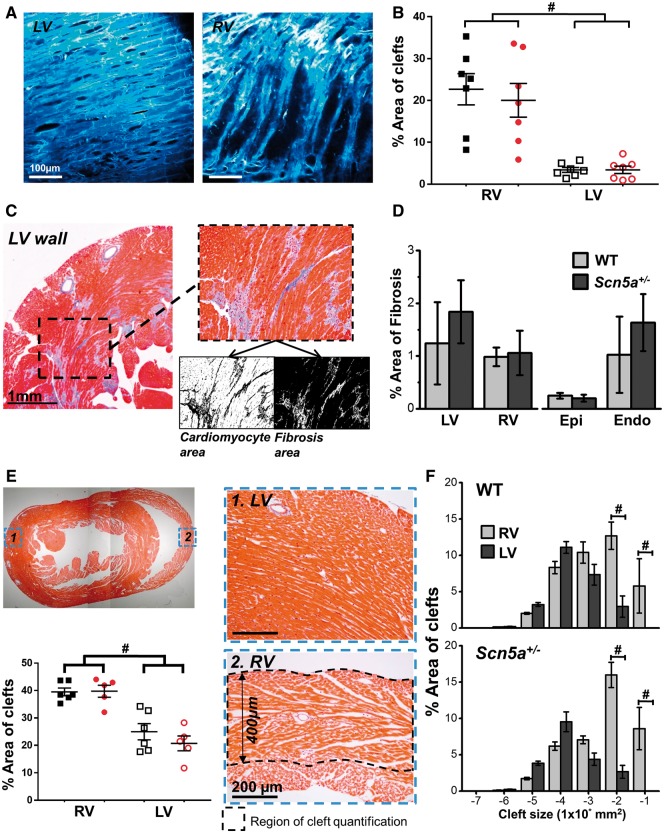
**  Transmural structural features in mouse LV and RV.** (*A*) Example images of cardiomyocyte density from 2 P imaging of LV (left) and RV (right) sub-epicardium (z-100 μm). (*B*) Quantification of non-vascular clefts from 2 P images, in WT (black squares, *n* = 7) and *Scn5a^+/−^* (red circles, *n* = 7) hearts. (*C*) Fibrosis quantification from histological section stained with Masson’s trichrome. Fibrosis is shown in blue. Black and white images below inset image show quantification of cardiomyocyte (left) and fibrosis (right) area using thresholding in ImageJ. (*D*) Left panel—% area of fibrosis from the transmural wall for both LV and RV. Right panel—% area of fibrosis measured from the LV sub-epicardium (from surface to deepest scan depth using 2P microscope) and the midmyocardium to sub-endocardium (portion of ventricle not scanned using two-photon). A total of eight serial sections were quantified for each heart and a single mean value obtained from these values. (*E*) Top left image shows a histological section stained with Masson’s trichrome, images on right indicate typical sections from 1. LV and 2. RV transmural wall. Absolute depth of quantification for both LV and RV is indicated in 2. Bottom left panel indicates quantification of non-vascular clefts in both ventricles from histological sections of WT (black squares, *n* = 6) and *Scn5a^+/−^* (red circles, *n* = 5) hearts. (*F*) Comparison of graded cleft sizes as a percentage of total cleft area between RV (light grey) and LV (dark grey). Top panel; WT mouse hearts (*n* = 6), bottom panel; *Scn5a^+/−^* mouse hearts (*n* = 5). Panels B, E, and F, #*P *<* *0.01, RV vs. LV. Two-way ANOVA with Bonferroni multiple comparisons test.

Given the disparity in electrophysiological behaviour between the LV and RV of *Scn5a*^+/^^−^ hearts, we sought to uncover underlying structural differences that may explain these observations. Initial 2 P imaging revealed distinct structural features 50–150 µm below the epicardial surface between the LV and RV in both *Scn5a^+/^*^*−*^ and WT groups (*n* = 7 for both). Representative LV and RV sub-epicardial 2 P images are shown in *Figure 4A*. Image analysis revealed that a significantly larger proportion of the RV was occupied by non-vascular clefts compared with the LV in both *Scn5a^+/^*^*−*^ and WT hearts (*Figure 4B*). These observations were explored in more detail using transverse short-axis histological slices taken midway between apex and base and stained with Masson’s trichrome (representative images in *Figure 4C*) to quantify the degree of transmural fibrosis (stained blue). No significant difference in fibrosis levels were observed, either between groups or between ventricles within each group (*Figure 4D*). Non-vascular spaces were quantified from histology across the LV and RV freewall for the intramural region observable by 2 P microscopy, from the epicardial surface down to a depth of 400 µm (indicated in the lower right image of *Figure 4E*). At a high magnification, the diffuse strand-like architecture of the RV becomes apparent in contrast to the densely compacted LV myocardium. In line with the 2 P-derived image analysis, a significantly larger intramural area is occupied by non-vascular extracellular clefts in the RV vs. LV, with no differences between WT and *Scn5a^+/^*^*−*^ (lower left panel of *Figure 4E*). As shown in *Figure 4F*, grouping the percentage cleft area by absolute size reveals that LV and RV diverge from each other at the largest cleft sizes (1 × 10^−2^ and 1 × 10^−1^ mm^2^) in both WT and *Scn5a*^+/^^−^ hearts.

### 3.5 Acute reduction of gNa in the intact heart

**Figure 5:**
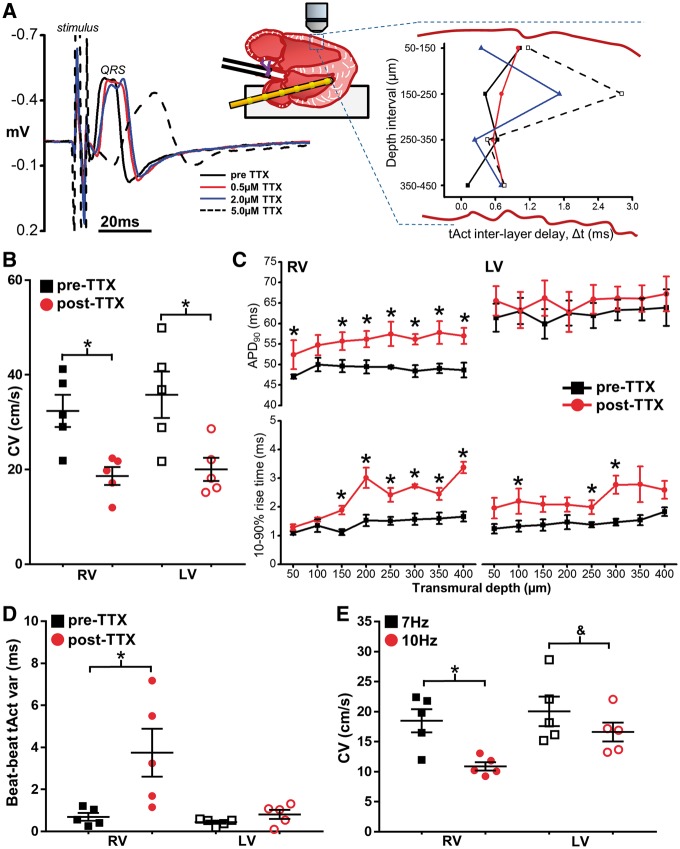
** Specific Na channel blockade in isolated hearts from healthy young mice.** (*A*) Acute blockade of Na channels in hearts from 12-week-old WT mice using TTX, resulted in a concentration-dependent prolongation of QRS duration on ECG (left panel) and an increase in inter-layer conduction delay heterogeneity (right panel). (*B*) RV and LV transmural CV before (pre-TTX) and after (post-TTX) perfusion of 2 µM TTX. (*C*) Transmural AP duration (top panels) and rise times (bottom panels) in each ventricle pre-TTX (black boxes) and post-TTX (red circles). (*D*) Mean beat-to-beat AP activation (tAct) variability in both ventricles pre-TTX and post-TTX perfusion. (E) CV in RV and LV post-TTX at two different stimulation frequencies. Panels B–E, *n* = 5. *P <* *0.05, difference *vs* pre-TTX perfusion (*B–D*), or 7 Hz vs. 10 Hz (*E*); ^&^*P *=* *0.05, 7 Hz vs. 10 Hz (*E*). Repeated measures two-way ANOVA with Bonferroni multiple comparisons test for panels B–E.

The observation that cleft size is different between LV and RV but not different between WT and *Scn5a*^+/^^−^ hearts suggests that a RV bias in the electrophysiological phenotype of Na-channel loss-of-function may result as a consequence of RV structure. To test this hypothesis and avoid the influence of confounding age-related changes, gNa was reduced acutely in isolated hearts from young (12 weeks old, *n* = 5) WT mice using the specific Na-channel inhibitor TTX. *Figure 5A* shows representative ECG recordings 20 min after perfusion with 0.5, 2.0, and 5.0 µM TTX, along with the resultant 2 P-derived transmural activation profiles at each concentration. In the presence of 5.0 µM TTX, tissue excitability diminished to such an extent that the voltage threshold for stimulation was increased substantially (>5 V) resulting in marked changes in epicardial activation patterns. At 2.0 µM, however, TTX reduced transmural CV in the LV and RV to 56.8 ± 7.4% and 57.3 ± 4.2%, respectively, of pre-treatment values (*Figure 5B*). TTX treatment significantly prolonged APD in the RV sub-epicardial and mid-myocardial layers but not the LV (*Figure 5C*, upper panels). Importantly, no transmural gradient in APD developed after TTX treatment over the 450 µm range investigated. Significant slowing of AP rise time also developed in both the RV and LV transmural wall in this subset of hearts following TTX perfusion (*Figure 5C*), an effect which was most pronounced in the RV. Most importantly, differences in transmural conduction and activation characteristics developed between LV and RV after TTX perfusion similar to those found in *Scn5a*^+/^^−^ hearts. Analysis of activation time from a train of APs captured with 2 P line scanning close to the epicardial surface revealed a significant increase in tAct variability in the RV, but not the LV, following TTX perfusion (*Figure 5D*). In addition, while increasing pacing frequency from 7  to 10 Hz after TTX perfusion slowed transmural CV in both ventricles (*Figure 5E*), the effect was greater in the RV vs. the LV, with CV decreasing by 36 ± 6% in the RV but only 16 ± 3% in the LV. Taken together, these results suggest that solely reducing gNa acutely via pharmacological inhibition is sufficient to reproduce many of the RV-specific conduction defects present in *Scn5a^+/^*^*−*^ hearts.

### 3.6 Computational modelling of transmural conduction

**Figure 6:**
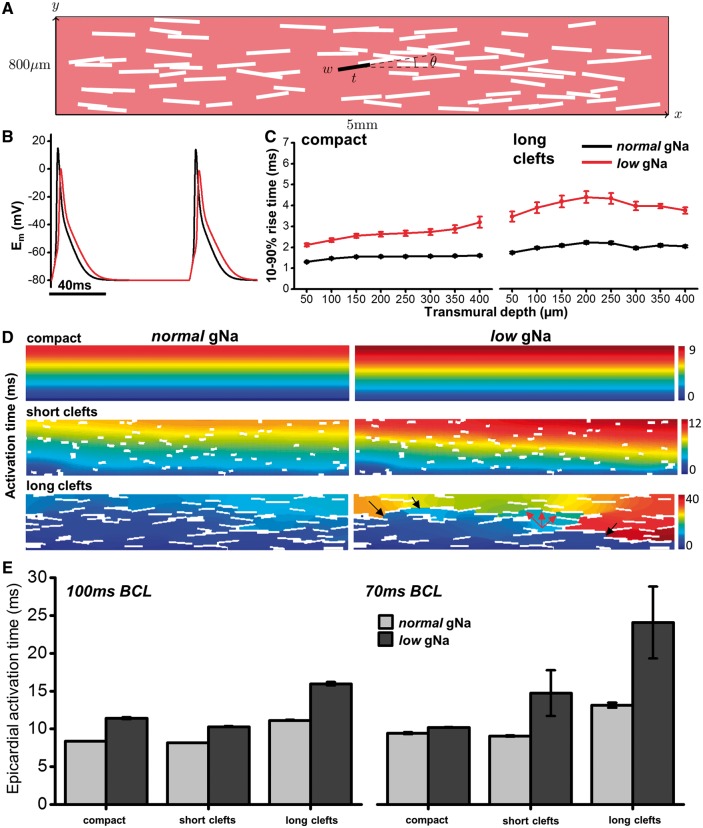
** Computational modelling of electrical propagation with structural discontinuities and reduced Na-channel conductance.** (*A*) Schematic of two-dimensional computational model incorporating randomly distributed non-conducting clefts with orientation described by *θ.* Cleft length was varied between ‘short’ and ‘long’ conditions. (*B*) APs generated from model with *normal* (black line) and *low* (red line) gNa**.** (*C*) AP rise times up to 400 µm from epicardial surface generated from the model under 100% (*normal*) and 50% (*low*) of regular gNa when tissue is compact (left panel) and in the long-cleft condition (right panel). (*D*) Colour maps of activation time in three different cleft conditions when gNa is *normal* (left panels) or *low* (right panels). Colour key shows activation time range in ms. Arrows in lower right panel indicate conduction slowing (red) and regions of conduction block (black). (*E*) Epicardial activation times for each cleft condition with *normal* (light grey) and *low* (dark grey) gNa are compared at 100 ms (left) and 70 ms (right) cycle lengths, indicating a sensitivity of *low* gNa to fast pacing rates in the presence of clefts. Bars indicate average activation time over 10 sequential tissue activations with mean ± standard deviation.

To quantify the influence of transmural structure on electrical conduction, a simplified 2 D model of transmural conduction was employed with varying degrees of structural heterogeneity (*Figure 6A*), mathematically described as non-conducting clefts, using either normal Na-channel conductance (*normal* gNa) or 50% of normal (*low* gNa). Representative APs under both conditions from the midwall of modelled tissue with no structural discontinuities (compact) are shown in *Figure 6B*. An increase in transmural AP rise times was apparent in the *low* gNa condition in the compact model (*Figure 6C*—left) which increased further using the largest proportion of structural discontinuities (*Figure 6C*—right). Transmural activation maps for several different model cleft configurations are displayed in *Figure 6D* for *normal* (left) and *low* (right) gNa. These maps demonstrate a prolonged transmural activation delay and increasing dispersion of activation with increasing cleft size when gNa is reduced by 50%. The consequence of tortuous conduction pathways that result under these conditions are illustrated in the lower right panel of *Figure 6D*. Red arrows indicate conduction slowing through the midwall region, while severe activation delay in later activated regions results in conduction block (black arrows). The influence of clefts in the presence of low gNa is also shown in Supplementary material online, Video S1. Activation delay at the epicardial surface is further amplified in the *low* gNa condition when basic cycle length is reduced from 100 ms (10 Hz) to 75 ms (∼13.3 Hz) (*Figure 6E*). Under these conditions, epicardial activation time is markedly increased, even when cleft size is short. Like the experimental results, prolongation of APD was also demonstrated in the model after reduction of gNa. APD_90_ over the first 400 µm of the model tissue is shown for compact (open symbols, dashed line) and long cleft (closed symbols) scenarios in Supplementary material online, *Figure S4 A*. In both cases, lower gNa results in APD prolongation throughout the transmural wall. The absolute magnitude of APD prolongation is, however, much higher in the presence of long clefts (Supplementary material online, *Figure S4B*), supporting the experimental findings of an RV-specific prolongation in APD when gNa is reduced.

## 4. Discussion

Loss-of-function of the cardiac sodium channel Na_V_1.5 is a typical feature of several genetic arrhythmic disorders. Attention has focused on BrS, initially described as an abnormality of electrical conduction among patients who survived SCD.^3^ Subsequent reports recognized and emphasized the role of subtle structural discontinuities due to increased intramural fibrosis in the generation of arrhythmias.^20^^,^^21^ Indeed, animal models of reduced Na-channel expression frequently demonstrate the presence of significant fibrotic tissue deposition with age.^22^^,^^23^ The current study now proposes an additional paradigm, whereby interaction between the electrophysiological consequences of reduced myocyte Na-channel expression and the normal structural discontinuities present in RV intramural tissue architecture predispose individuals to RV conduction abnormalities before the onset of increased fibrosis. Structural heterogeneity in the RV intramural wall results in tortuous conduction pathways for electrical propagation, making this tissue more vulnerable to activation delay, depolarization heterogeneity and conduction slowing relative to the more compact LV. This is supported by computational modelling and transmural electrophysiological data from hearts with acute reduction of I_Na_ using TTX. The optical sectioning capability of 2P imaging allowed exploration of transmural structure and electrophysiological function not possible with either optical mapping or surface electrode arrays alone, whilst preserving the electrical syncytium of the heart. The precision afforded by this technique revealed subtle changes in transmural CV through the RV mid-myocardial and sub-epicardial layers during rapid pacing when Na-channel function is impaired. Further developments to this technique currently underway, including high sensitivity red-shifted fluorophores and increased light collection, will improve the optical sectioning at depth and enhance the utility of 2P imaging for more advanced deep tissue electrophysiological studies.

### 4.1 The role of tissue architecture in ventricular conduction

Cardiac structural abnormalities are an important risk factor for arrhythmias and SCD^24^; however the mechanisms by which structural discontinuities predispose individuals to arrhythmias remain incompletely understood. For an abrupt tissue expansion (thin tissue strand leading to expanded region of myocardium), current drain owing to increased electrotonic loading of the expansion reduces the safety for propagation. In the setting of reduced Na current this can lead to a marked slowing, or even blockade of conduction in that region.^25^^,^^26^ Source–sink mismatch produced by abrupt tissue expansions may also promote early after depolarizations, via persistent Na current, resulting in impulse reflection and re-entry.^24^ These mechanisms have previously been thought relevant primarily in structural heart disease such as scar formation following myocardial infarction, or the plethora of fibrotic cardiac diseases, which are often additionally accompanied by cardiomyocyte remodelling.^27^ The key finding of this study, however, is that under conditions of reduced Na channel expression/function, no structural abnormality need be present to explain an increased propensity to conduction disturbance in the RV. The more discontinuous, albeit structurally normal RV architecture predisposes individuals to further conduction defects, relative to the LV. Furthermore, this effect can be demonstrated acutely using the highly specific Na channel inhibitor TTX in healthy hearts with no cellular remodelling.

The abundance of transmural extracellular clefts between WT and *Scn5a*^+/^^−^ hearts was similar in the present study, indicating that reduced expression of Na_V_1.5 does not influence ventricular structural development. Furthermore, our results suggest there is a higher tolerance to reduced Na channel activity in the compact LV, which exhibited less beat-to-beat (2 P measurements) and spatial (computational model) activation heterogeneity compared with the RV, despite transmural CV in *Scn5a*^+/^^−^ hearts being similarly reduced in both ventricles. Acute Na channel inhibition in WT hearts with TTX (reducing CV to levels comparable to *Scn5a*^+/^^−^ hearts) qualitatively recapitulated the differential transmural conduction behaviour between RV and LV seen in *Scn5a^+/^*^*−*^ hearts. The TTX concentration used was close to the previous reported *K*_d_ (1.6 µM) based on I_Na_ binding curves.^28^ These data support the notion that intrinsic interventricular structural disparity is a dominant factor predisposing the RV to heterogeneous conduction and arrhythmogenesis in the face of reduced Na channel abundance.

To the best of our knowledge, an interventricular comparison of non-vascular clefts has yet to be made. LeGrice *et al.*^29^ performed a comprehensive characterization of the laminar organization of canine LV and RV, noting the appearance and regional variation in angle of cleavage planes present throughout the transmural space, however a direct LV-RV comparison was not reported. Pope *et al.* also performed a detailed three-dimensional examination of transmural laminar arrangement in rat LV and found the sub-epicardial layers to be compact with relatively fewer clefts compared with more discontinuous mid-wall, a finding closely resembling the LV structure seen from 2P imaging and short-axis histological images in the current study.^30^ Based on the 2P sub-epicardial images, it appears that the most marked structural differences between ventricles lies within this region, however, a similar analysis of RV transmural structure was not performed in the study by Pope *et al.* to fully address this. Consequently, we believe that this study is the first to investigate the potential influence of interventricular transmural cleft size and distribution on arrhythmia susceptibility in a model of reduced Na channel expression.

Activation heterogeneity and increased conduction slowing in RV vs. LV results from tortuous conduction pathways, which depend not only on the total volume of clefts within the intramural wall but also on the frequency of coupling between groups of myocytes. While this is evident to an extent from the short-axis histological slices, this was not explicitly quantified from experimental data. The influence of the coupling frequency is examined in more detail, however, using the computational model, by varying the length of the intramural clefts (rather than area). This more closely approximates the laminar cleft orientation and volume seen experimentally and allowed this aspect to be explored in a controlled manner. Another important aspect is fibre orientation; myocyte layers are known to exhibit pronounced fibre rotation between the endo and epicardial surfaces.^29^ Using only short-axis histological images, we were not able to fully resolve the extent of transmural fibre rotation. While anisotropy is an aspect of cellular electrophysiology known to influence conduction velocity, the electrical propagation axis remains normal to the cardiomyocyte long axis throughout, likely minimizing the influence of fibre rotation.

Of note is the influence of clefts on APD. In both experimental groups, when Na channel function was reduced, RV APD was prolonged relative to control while LV APD was unaltered. The model demonstrated that in compact tissue (analogous to LV), APD was prolonged only slightly (∼5 ms), but the presence of long clefts (analogous to the RV) increased the magnitude of APD prolongation by ≈2×. Both experiments and model therefore suggest a significantly greater influence of reduced gNa (or Na channel expression) on transmural repolarization in the presence of structural heterogeneities such as those within the RV wall. The change in APD from compact tissue observed in the model was not seen experimentally in the LV, but this could be attributed to the relatively small change being masked by variation in APD between experimental preparations as well as variability associated with the method of measurement. Modelling transmembrane potentials across a region of tissue equivalent to the 2 P experimental imaging (400 µm) revealed a transmural APD gradient between 4 and 5 ms (see Supplementary material online, *Figure S4 A*; compact and long clefts, respectively) under low gNa conditions. This may have been too small to detect reliably over this range of tissue depths.

### 4.2 Structure vs. ion channel heterogeneity

Several aspects of ventricular ion channel heterogeneity have been proposed to explain the increased RV susceptibility to arrhythmias in the presence of Na channel loss-of-function mutations. Expression of Na_V_1.5 has been reported to be lower in the RV of Scn5a^+/^^−^ mice.^11^ However, in the same study, the RV-LV difference in Na-channel protein expression in WT animals was much less (<10% vs. ∼30% in Scn5a^+/^^−^ mice).^11^ Here, we show that when peak Na current in young WT hearts is reduced equally in both ventricles using TTX, the RV remains more susceptible to conduction abnormalities compared to the LV. Mouse ventricles also exhibit higher subendocardial Na-channel abundance relative to the subepicardium.^31^ Previous work from our group indicated that in intact ventricular tissue electrotonic current drain decreases as a normally conducted wave-front approaches the epicardial surface, resulting in increased AP upstroke velocity relative to the deeper layers.^16^ Importantly, this effect occurs despite the epicardium exhibiting a lowered peak Na current. Therefore, lower subepicardial Na-channel expression would not necessarily be expected to negatively influence epicardial conduction in the Scn5a^+/^^−^ mouse. In line with this observation, RV rise time from both *Scn5a*^+/^^−^ hearts and TTX-treated hearts was not different from that of controls close to the epicardium (*Figure *1*D* and 5C, respectively). In ventricular tissue of limited size, such as the mouse, no functional APD dispersion is seen across the LV transmural wall due to electrotonic coupling, even when allowing for transmural gradients in I_Na_ and I_to_.^14^ This finding is supported by previous experimental measurements.^32^ In the present study, the use of computational modelling tested solely the influence of structural heterogeneities on electrical conduction without the confounding transmural variability in ion channel expression (*Figure 6*). These data support the conclusion that structural heterogeneities alone are enough to influence conduction delay and heterogeneity, particularly at increased heart rates.

The unique developmental origin of the RVOT has emerged in recent years as an explanation for the increased RV susceptibility to arrhythmias (for a comprehensive review, see Boukens *et al.*).^33^ In mice, the RVOT retains a foetal gene pattern almost until birth and in adults displays slower conduction than the RV free wall both after treatment with the Na channel blocker ajmaline, and in hearts heterozygous for a BrS-linked Scn5a mutation (*Scn5a*^1798insD/+^)^34^ suggesting the residual embryonic phenotype of the RVOT explains the increased propensity for arrhythmias to arise there. We sought to isolate as far as possible the influence of normal, non-fibrotic structural discontinuities by studying conduction in the RV and LV free wall distinct from the RVOT. While the RVOT represents a vulnerable structure in BrS for the reasons discussed earlier, we suggest that conduction abnormalities can also arise in structurally normal RV tissue when Na channel function is depressed due to the presence of intramural clefts. Although not examined in this study, this structure-based explanation for conduction abnormalities could be applied to the more fibrotic tissue of the RVOT and thus may confer even greater vulnerability to conduction block in the RVOT compared to the surrounding RV free wall.

### 4.3 The importance of interstitial fibrosis versus intramural tissue clefts

Interstitial fibrosis has been reported among patients with BrS,^20^^,^^21^ particularly in the RVOT. In contrast, the results using the Scn5a^+/^^−^ mouse have been mixed, with increased fibrosis of both ventricles noted in some,^23^^,^^35^ and preference for greater levels of LV fibrosis vs. RV, for others.^22^^,^^36^ The current study found no significant difference in RV or LV fibrosis of *Scn5a*^+/^^−^ hearts vs. WT. The reason for this difference is unclear but might be related to the age of the animals studied and/or the penetrance of the conduction disorder. The onset of marked fibrosis is age-dependent^35^^,^^37^ with some studies reporting data from mice aged older than 70 weeks.^22^^,^^35^ In the present study, 5/8 *Scn5a*^+/^^−^ mice were 60 weeks old, possibly representing varying degrees of transition into profibrotic states. Worsening fibrosis undoubtedly plays a role in the progression of conduction defects with age in the Scn5a^+/^^−^ mouse model; however, this mouse also demonstrates increased susceptibility to ventricular arrhythmias from a young age (8–36 weeks), when fibrosis would not be expected to play an appreciable role.^7^^,^^15^ Recently, optical mapping of a porcine model expressing an *SCN5A* loss-of-function mutation found in BrS patients revealed an increased propensity for both pacing induced and spontaneous VF, often originating from the RV free wall.^38^ Crucially, there was no evidence of intramural fibrosis in any region of the heart from these animals, even up to 2 years of age. These observations support the notion that intramural fibrosis is an exacerbating factor but not a requirement for the appearance of lethal arrhythmias when Na channel function is reduced.

### 4.4 Study limitations

Transmural electrophysiological data were acquired sequentially as simultaneously examining many layers in intact tissue using 2P imaging was not possible, limiting analysis to steady state behaviour synchronized to the stimulus pulse. Nevertheless, it enabled average transmural conduction behaviour of both ventricles to be examined within the same preparation with minimal physical tissue disruption, maintaining the preparation as close to physiological as possible. Ventricular wedge preparations are routinely used for optical interrogation of transmural electrophysiology^39^; however, the small size of the mouse heart precludes use of such an approach.

Computational modelling of the ventricular wall in 2 D utilized a verified mouse ventricular cell model to mirror the intact mouse heart used experimentally. The electrophysiological characteristics of the mouse AP are different from humans and other larger mammals, which could introduce disparities when trying to extrapolate results to larger species. To assess the contribution of species differences, parallel simulations were performed using the Luo-Rudy guinea pig ventricular cell model that recapitulates many of the electrophysiological characteristics of human cardiomyocytes.^40^ The results qualitatively agreed with those using the Bondarenko^19^ mouse model (data not shown), highlighting the same fundamental mechanism related to conduction slowing and tortuous activation pathways in the setting of intramural discontinuities and reduced gNa. Clefts were modelled as non-conducting voids to simplify the computational process despite these regions in the whole heart containing extracellular fluid (Tyrode’s solution in *ex vivo* experiments). This approach was justified, as previous simulations using the full bidomain representation with a highly conducting medium in the cleft produced almost identical results (data not shown). 2 D modelling might be expected to exaggerate phenomena compared to a more realistic 3 D model (due to lower overall coupling and electrotonic load). The 2 D model in this study was employed primarily to gain mechanistic insight into the role of structure rather than replicate the experimental results. For this purpose, the 2 D representation was more than sufficient. A representative 3 D model requires greater detail of cleft frequency in both short and long axis transmural planes, something that was not possible using histological sections in the current study. Future studies may look to construct a similar model in 3 D using 2P imaging of optically cleared intact hearts. This approach would also aid in mapping the measured electrical activity directly onto the corresponding structure, something that was not possible using the histological sections without physical fiduciary markers.

## 5. Conclusions

We demonstrate that normal structural heterogeneities present in the RV are responsible for increased vulnerability to conduction slowing in the presence of reduced Na channel function. Indeed, the greater density of transmural clefts in the RV was sufficient to explain the distinct electrophysiological phenotype without the need for additional fibrosis. This heterogeneous conduction slowing will predispose to functional block and the initiation of re-entry, and as such may explain the increased susceptibility to VF in BrS, a condition frequently characterized by loss-of-function Na channel mutations and associated conduction slowing specific to the RV.

## Supplementary material

Supplementary material is available at *Cardiovascular Research* online.

## Supplementary Material

Supplementary Data
