# Supplementary material for: Normal interventricular differences in tissue architecture underlie right ventricular susceptibility to conduction abnormalities in a mouse model of Brugada syndrome
Source: Cardiovasc Res. 2017 Dec 18;114(5):724–36. doi: 10.1093/cvr/cvx244 (PMC5915948; doi:10.1093/cvr/cvx244)
Supplement: Supplementary Data [file cvx244_supp.zip › cvx244-suppl_data/Supplementary material_final.docx]

**SUPPLEMENTARY MATERIAL**

**Expanded Methods**

**Isolated heart preparation**

A total of 26 mice were used in the study. For the initial phase, 11 *Scn5a^+/-^* and 10 aged-matched WT littermates were compared. A further 5 WT mice (12 weeks old) were used to investigate the effects of acute TTX perfusion. Animals were sedated with a gaseous mixture of 2% isoflurane in 70/30 N_2_O and O_2_. Under deep anesthesia the chest was opened and 0.05ml of 1000IU/ml heparin was injected into the inferior vena cava to minimize clotting. After excision, the heart was placed in cold (4ºC) Tyrode’s solution and transferred to a constant flow perfusion system where the aorta was cannulated, and the heart was perfused with oxygenated 37°C Tyrode’s solution containing (mmol/L) NaCl (116), NaHCO_3_ (20), Na_2_HPO_4_ (1), MgSO_4_ (1), KCl (5), CaCl_2_ (1.5), Glucose (11), Na-pyruvate (1.2). The time from excision to cannulation was kept to under 3 min. Flow was adjusted to maintain a steady perfusion pressure of 60-80mmHg throughout, recorded using an inline pressure sensor (ADInstruments; Oxford, UK). Bath temperature was monitored throughout with a temperature probe. An incision was made in the left atrial auricle and a 1.2F Scisense octapolar electrophysiology catheter (Transonic; Ithaca, NY) was advanced into the LV and positioned at the apex. The most distal electrode was used to stimulate the heart. Pacing was controlled using a Myopacer stimulator unit (Ionoptix; Westwood, MA). A volume-conducted ECG was recorded throughout by placement of 4mm Ag/AgCl disc electrodes around the heart in a standard lead I configuration. After a 15-min equilibration period perfusion was switched to a Tyrode’s solution containing a combination of blebbistatin (10µmol/l) and 2, 3-butanedione monoxime (5mmol/l) to suppress contraction throughout the experiment. Hearts were loaded with di-4-ANEPPS (25µl of 2mmol/l stock, diluted in 1ml Tyrode’s solution) for voltage measurement, using a slow bolus injection protocol over a period of 10-15min. A schematic diagram of the isolated heart setup is shown in Supplementary figure 1A.

**Optical action potential recordings**

Whole heart fluorescence experiments were performed using a custom-built ViVo two-photon microscope system (Intelligent Imaging Innovations; Denver, CO), with a 128x128 pixel CardioCMOS-SM128 camera (Reshirt Imaging; Decatur, GA) and 120fs pulsed Ti-sapphire two-photon laser (Coherent; Santa Clara, CA) attached. Widefield epifluorescence imaging was performed using a 2.5X 0.3NA air objective with a 470nm LED excitation source passed through the objective via a side port light path on the microscope system. Emitted light was filtered through a 590nm long pass external filter. For two-photon recordings, a 20X 1.0NA water-dipping objective (working distance 1.8mm) was used. Emitted fluorescence collected through the lens was directed through a short pass filter (650nm), split with a dichroic mirror and directed onto two high sensitivity GaAsP PMT detectors; shorter wavelength light (525-560nm) was then focused onto PMT1, and longer wavelength light (>590nm) onto PMT2. For di-4-ANEPPS, an excitation wavelength of 920nm was used and a ratio of the resultant signals from both PMTs was taken. For both ventricles, starting with the RV, the preparation was positioned with the ventricular freewall halfway between apex and base, approximately in the centre of the field of view. Optical maps were recorded initially at 7Hz stimulation to ensure activation pattern was consistent with transmural (endo-epicardial) activation. Two-photon measurements were then taken after identifying the absolute epicardial surface; the interface between the connective tissue surface layer and the underlying myocardium. Line scan sequences (4s duration per scan) were acquired serially from 450µm to 50µm below the epicardial surface in 50µm steps. Laser intensity was automatically adjusted to avoid saturation at the shallow layers where the fluorescent signal was brightest. Line scans were oriented along the longitudinal axis of cells at the surface and corresponded to 200µm in length (approximately 2 cell lengths). After the z-stage returned to its original position images were captured to confirm the preparation had not moved. Scan sequences were rejected and reacquired if the preparation was >25µm away from its starting position.

**Stimulus methodology for transmural activation pattern**

To ensure ventricular activation and transmural conduction were as close to physiological as possible whilst maintaining the heart in an intact state, 4 isolated heart preparations (2 WT and 2 *Scn5a*^+/-^) were additionally paced from the right atrium at 7Hz to produce a physiological activation pattern, and transmural conduction velocity compared with stimulation at the endocardial LV apex. While the most physiological activation pattern was desirable, it was not possible for two reasons: (i) rapid pacing protocols, used to test the limits of ventricular transmural conduction, if initiated from the right atrium would be limited by the atrioventricular (AV) nodal conduction and not by the intrinsic limit of the ventricular tissue being compared, and (ii) the pan heart nature of reduced Na channel expression in the *Scn5a*^+/-^ mouse would further limit AV nodal conduction relative to WT hearts, introducing a further bias which would complicate interpretation. As shown in Supplementary figure 2A, conduction velocity for these 4 preparations was not changed when pacing from either site, while epicardial activation time was significantly higher while pacing from the right atrium due to the longer conduction path for a propagating stimulus (Supplementary figure 2B). Pacing was therefore performed from the LV endocardium.

**Tissue histology**

At the end of isolated heart experiments, hearts were immediately cut from the aortic cannula and placed in light-shielded tubes with phosphate-buffered saline solution containing 4% paraformaldehyde, and stored at 4ºC. After two weeks, hearts were embedded in a paraffin block and cut in the horizontal plane at the level of the ventricular midwall. A series of 8 non-sequential, 4µm thick slices were taken of each heart sample; One slice was taken, followed by 5 slices which were discarded, then the next slice taken, etc. Data points for histological parameters in each heart represent a mean of these 8 image slices.

**Data analysis**

ECG, temperature, pressure, stimulus pulses and capture triggers (from CMOS camera and laser scanning microscope, respectively) were digitized using an 8-channel Powerlab A/D board running Labchart 6.1 (ADInstruments; Oxford, UK) and recorded to a PC.

***Optical imaging***

Widefield optical mapping data were analyzed using Optiq AP analysis software (Dr Francis Burton, University of Glasgow, UK / Cairn Research – Faversham, UK). Two-photon derived voltage signals were analyzed separately using custom-written software in Matlab and Delphi. To ensure adequate signal/noise ratio for signals captured at deeper layers, a train of 20 APs were averaged at each layer.

***Histological image analysis***

The degree of fibrosis was quantified using a modified version of a color thresholding method in ImageJ, compatible with histological images stained with Masson’s trichrome^1^. The tissue compartments of interest were assigned color thresholds based on empirically-derived values from a test set of images. For each region within the image, the luminal portion was identified and subtracted as a background component, leaving total tissue component. Based on the color threshold preassigned to tissue (stained red) and fibrosis (blue), the % of each as a total of the remaining tissue component was calculated, first by identifying the fibrotic region, then subtracting this from the remaining tissue component. Fibrosis was quantified for the entire free wall of the LV and RV. This was then further split into an epi region, representing the area of the free wall measurable with two-photon line scanning (up to 450µm from the absolute epicardial surface) and an endo region, representing the remaining free wall. Example images are shown in Figure 4C.

For intramural clefts, an ImageJ particle analysis module was used. The selected image region was converted to a binary image where non-vascular gaps appeared in black and the remainder of the tissue in white. For histological sections, only the epi region, as defined above was included in the analysis. To avoid the larger endocardial structures (papillary muscles, trabeculations etc.) the volume measurable with two-photon line scanning was used as it becomes increasingly difficult to distinguish true intramural clefts versus endocardial structures using histological sections alone. For two-photon confocal images, a frame scan 100µm below the epicardial surface was captured and analyzed in the same manner as above.

**References**

1. Hadi AM, Mouchaers KT, Schalij I, Grunberg K, Meijer GA, Vonk-Noordegraaf A, van der Laarse WJ, Beliën JA. Rapid quantification of myocardial fibrosis: a new macro-based automated analysis. *Cellular Oncology*. 2011;34:343–354.

**Supplementary figure legends**

**Supplementary figure 1. Fluorescent imaging of the isolated mouse heart. A,** schematic of the experimental setup. **B,** left shows a typical raw image obtained from a CMOS camera showing a portion of the left ventricle free wall using a 2.5x magnification air objective, next to a schematic drawing of the analysis region with AP traces of the red spectrum of the di-4-ANEPPS emitted fluorescence. Left atrium is indicated with an arrow. Right illustrates typical AP recordings from 2P line scans at different transmural layers using a 20x 1.0NA water immersion objective. LA and RA, left and right atrium; LV and RV, left and right ventricle.

**Supplementary figure 2. Influence of pacing site on transmural conduction velocity.**  2P data from 4 isolated heart preparations (2 WT, black squares; 2 *Scn5a^+/-^*, red circles) showing left ventricular (LV) transmural conduction velocity (**A**) and epicardial activation time (**B**) when pacing from LV apex or the right atrium. Conduction velocity was not different when pacing from either site, while time from stimulation to epicardial activation was predictably much longer when pacing from the right atrium. **P*<0.05, paired Student’s t-test.

**Supplementary figure 3. Electrocardiograms from the Scn5a^+/-^ mouse. A,** representative *in vivo* ECG recordings from WT (left panel) and *Scn5a^+/-^* (right panel) mice at 10 weeks of age. Expanded insets indicate longer QRS duration in *Scn5a^+/-^* mice. **B**, ECG parameters from mice at 10 weeks of age (left panel) and from isolated hearts during Langendorff perfusion (right panel). **P*<0.05 *Scn5a^+/-^* *vs* WT. Statistical comparison performed using unpaired Student’s t-test.

**Supplementary figure 4. Influence of tissue structure on APD prolongation after reduced gNa in mouse tissue model. A**, Transmural APD_90_ values under normal gNa (black squares) and low gNa (red circles) in compact tissue (open symbols), and tissue exhibiting long clefts (closed symbols). Values are mean and standard deviation of 10 consecutive model tissue stimulations. **B**, mean difference in APD change (ΔAPD) after lowering gNa in compact (black squares) and long cleft (red circles) model tissue.
