# Supplementary figures and images for: Normal interventricular differences in tissue architecture underlie right ventricular susceptibility to conduction abnormalities in a mouse model of Brugada syndrome
Source: Cardiovasc Res. 2017 Dec 18;114(5):724–36. doi: 10.1093/cvr/cvx244 (PMC5915948; doi:10.1093/cvr/cvx244)

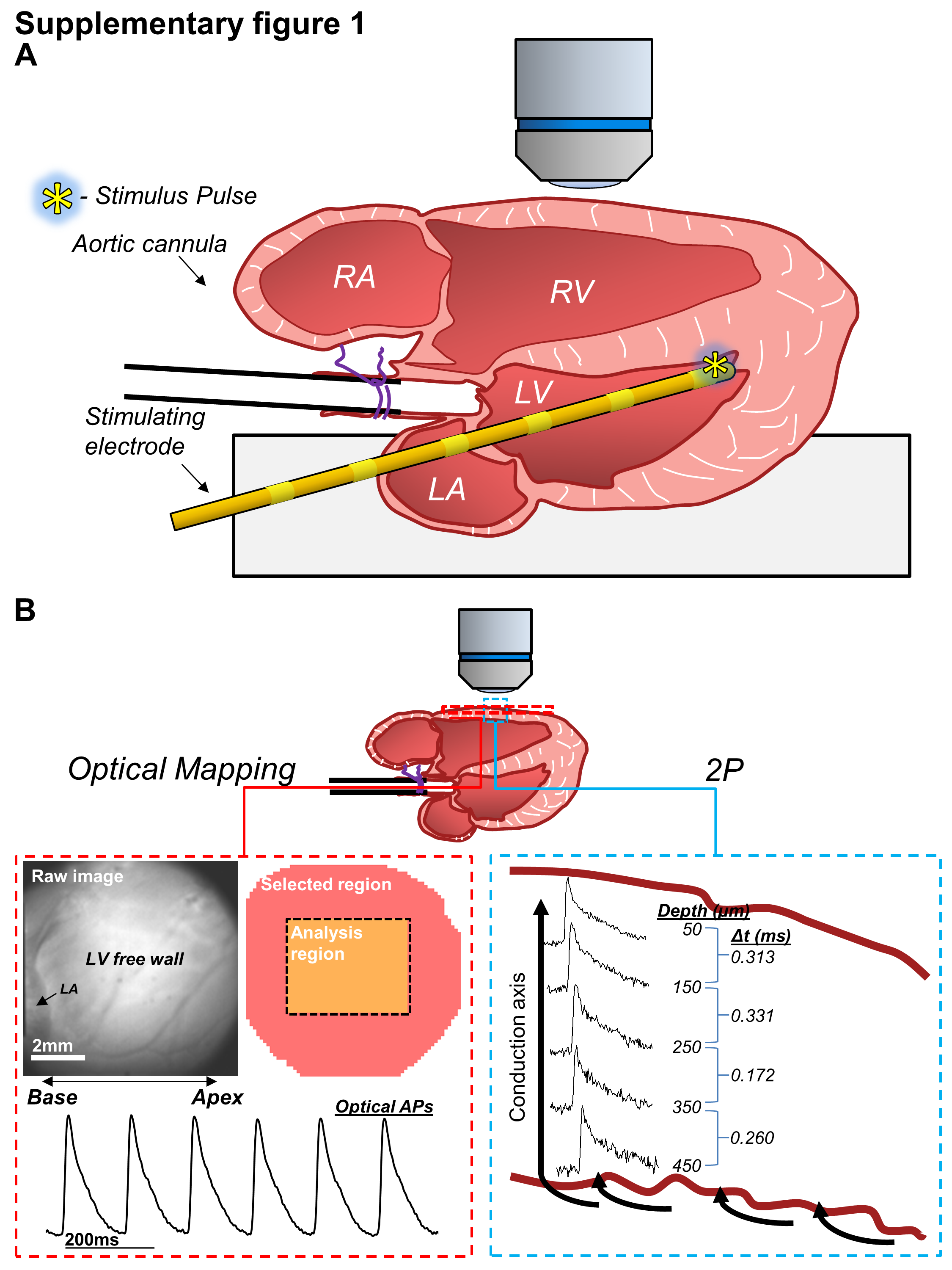

Supplement: Supplementary Data [file cvx244_supp.zip › cvx244-suppl_data/Supp fig 1.tif]

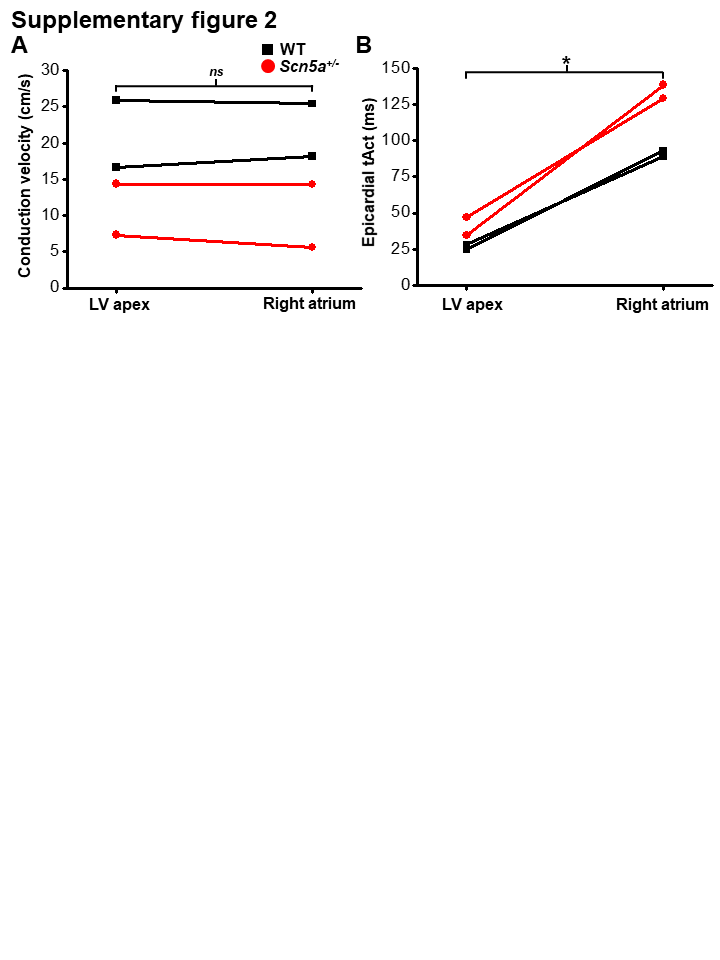

Supplement: Supplementary Data [file cvx244_supp.zip › cvx244-suppl_data/Supp fig 2.tif]

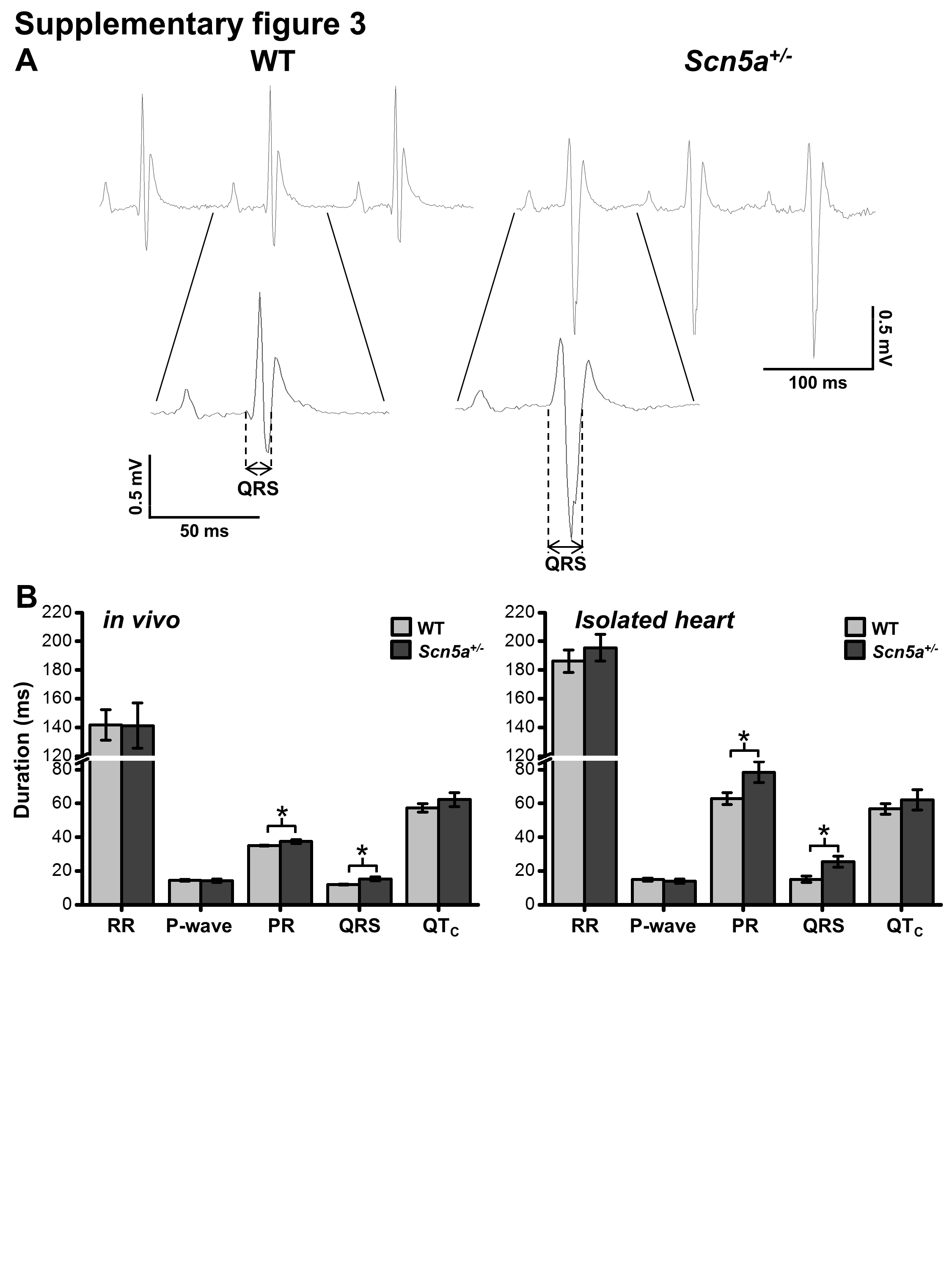

Supplement: Supplementary Data [file cvx244_supp.zip › cvx244-suppl_data/Supp fig 3.tif]

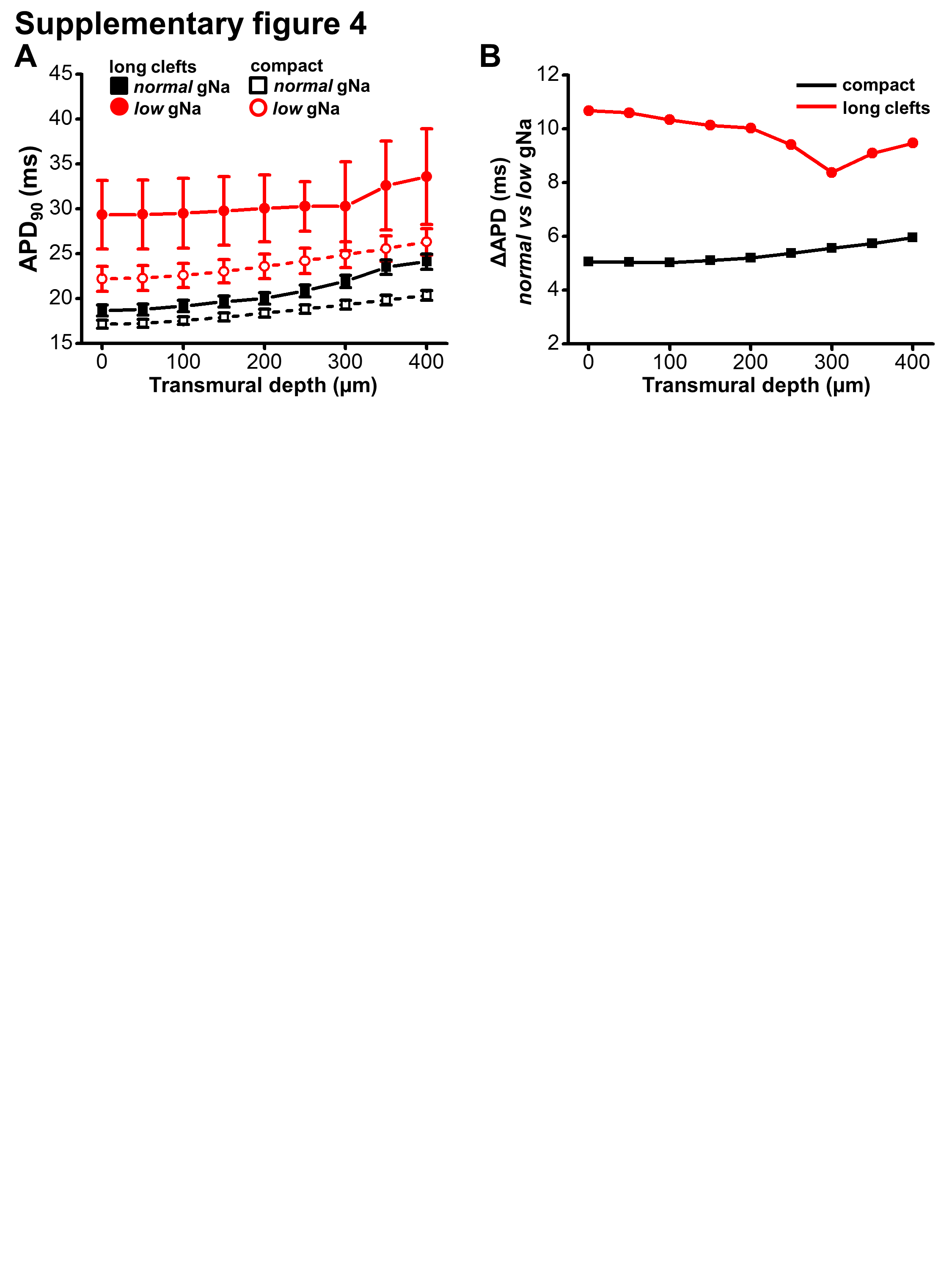

Supplement: Supplementary Data [file cvx244_supp.zip › cvx244-suppl_data/Supp fig 4.tif]
